# Typhoid Fever

**Published:** 1887-06

**Authors:** G. W. Sherbino

**Affiliations:** Abilene, Texas


### TYPHOID FEVER.

### BRY., ALB.$^{cm}$. (FINCKE).

G. W. SHERBINO, M. D., ABILENE, TEXAS.

Boy, æt. fifteen; had been going to school; was taken with fever. I saw him on twelfth day. He had not taken any drugs.

Delirious. Talking and muttering. Eyes wide open. Had not slept any for forty-eight hours. Wants to get out of bed and go home (Bell. Opium.) *He is driving oxen or repeating lessons. Mind runs on what he had been doing for past few weeks.* Tongue coated brown; crack at the tip running back for half-inch. Tongue, teeth, lips, covered with sordes; slight subsultus tendinum; two allopaths saw the case and gave an unfavorable prognosis. He received one dose at 8 A. M.; soon went to sleep, and delirium soon passed away. In three or four days he grew worse; one more dose was all that was needed.

